# Genome-Wide Analyses of Nephrotoxicity in Platinum-Treated Cancer Patients Identify Association with Genetic Variant in *RBMS3* and Acute Kidney Injury

**DOI:** 10.3390/jpm12060892

**Published:** 2022-05-28

**Authors:** Marije J. Klumpers, Ward De Witte, Giovanna Gattuso, Elisabetta Schiavello, Monica Terenziani, Maura Massimino, Corrie E. M. Gidding, Sita H. Vermeulen, Chantal M. Driessen, Carla M. van Herpen, Esther van Meerten, Henk-Jan Guchelaar, Marieke J. H. Coenen, D. Maroeska W. M. te Loo

**Affiliations:** 1 Department of Pediatrics, Radboud University Medical Center, Postbox 9101, 6500 HB Nijmegen, The Netherlands; marije.klumpers@radboudumc.nl; 2 Department of Human Genetics, Radboud University Medical Center, Postbox 9101, 6500 HB Nijmegen, The Netherlands; ward.dewitte@radboudumc.nl (W.D.W.); marieke.coenen@radboudumc.nl (M.J.H.C.); 3 Pediatric Oncology Unit, Fondazione IRCCS Istituto Nazionale dei Tumori, Via Giacomo Venezian, 1, 20133 Milan, Italy; giovanna.gattuso@istitutotumori.mi.it (G.G.); elisabetta.schiavello@istitutotumori.mi.it (E.S.); monica.terenziani@istitutotumori.mi.it (M.T.); maura.massimino@istitutotumori.mi.it (M.M.); 4 Princess Maxima Center for Pediatric Oncology, Postbox 113, 3720 AC Bilthoven, The Netherlands; c.e.m.gidding@prinsesmaximacentrum.nl; 5 Department for Health Evidence, Radboud University Medical Center, Postbox 9101, 6500 HB Nijmegen, The Netherlands; sita.vermeulen@radboudumc.nl; 6 Department of Medical Oncology, Radboud University Medical Center, Postbox 9101, 6500 HB Nijmegen, The Netherlands; chantal.driessen@radboudumc.nl (C.M.D.); carla.vanherpen@radboudumc.nl (C.M.v.H.); 7 Department of Medical Oncology, Erasmus MC Cancer Institute, Postbox 2040, 3000 CA Rotterdam, The Netherlands; e.vanmeerten@erasmus.nl; 8 Department of Clinical Pharmacy & Toxicology, Leiden University Medical Center, Postbox 9600, 2300 RC Leiden, The Netherlands; h.j.guchelaar@lumc.nl

**Keywords:** cisplatin, carboplatin, nephrotoxicity, acute kidney injury, hypomagnesemia, GWAS, *RBMS3*

## Abstract

Nephrotoxicity is a common and dose-limiting side effect of platinum compounds, which often manifests as acute kidney injury or hypomagnesemia. This study aimed to investigate the genetic risk loci for platinum-induced nephrotoxicity. Platinum-treated brain tumor and head–neck tumor patients were genotyped with genome-wide coverage. The data regarding the patient and treatment characteristics and the laboratory results reflecting the nephrotoxicity during and after the platinum treatment were collected from the medical records. Linear and logistic regression analyses were performed to investigate the associations between the genetic variants and the acute kidney injury and hypomagnesemia phenotypes. A cohort of 195 platinum-treated patients was included, and 9,799,032 DNA variants passed the quality control. An association was identified between *RBMS3* rs10663797 and acute kidney injury (coefficient −0.10 (95% confidence interval −0.13–−0.06), *p*-value 2.72 × 10^−8^). The patients who carried an AC deletion at this locus had statistically significantly lower glomerular filtration rates after platinum treatment. Previously reported associations, such as *BACH2* rs4388268, could not be replicated in this study’s cohort. No statistically significant associations were identified for platinum-induced hypomagnesemia. The genetic variant in *RBMS3* was not previously linked to nephrotoxicity or related traits. The validation of this study’s results in independent cohorts is needed to confirm this novel association.

## 1. Introduction

Platinum agents have been widely used as chemotherapeutic agents in a multitude of malignancies for decades. Cisplatin (*cis*-diamminedichloroplatinum) was introduced in the 1970s, followed up by the analog carboplatin (*cis*-diammine(1,1-cyclobutanedicarboxylato)platinum) a decade later [1]. Platinum agents derive their cytostatic effect by binding to nucleophilic sites of the DNA, forming crosslinks and inhibiting DNA synthesis, resulting in arrested replication and the death of proliferating cells [2,3]. Carboplatin, containing a cyclobutene ring instead of the two chloride ions of cisplatin, is considered a more stable and less reactive compound, making it less potent regarding both efficacy and toxicity compared to cisplatin [4,5]. Currently, both agents are cornerstones of the chemotherapeutic regimens used against a broad spectrum of solid tumors in children and adults. As with all chemotherapeutics, several toxicities occur due to the drugs’ poor selectivity of tumor cells over normal healthy cells [6].

Nephrotoxicity is one of the dose-limiting side effects of platinum agents, especially in older patients [7]. It occurs because of the renal excretion of platinum agents through both glomerular filtration and tubular secretion, resulting in high concentrations of the drugs within the kidneys [8,9]. The active transport of platinum compounds over the basolateral membrane, in combination with biotransformation, which increases its toxic potency, leads to the accumulation of platinum compounds in renal tubule cells, where their cytotoxic effect results in cell injury and cell death [10,11,12]. Additional local inflammatory responses and ischemic damage due to the vasoconstriction of the renal vasculature further increase renal toxicity [13,14]. The most common clinical presentation of these mechanisms is acute kidney injury (AKI), which manifests as an increase in serum creatinine and renal electrolyte wasting, most predominantly magnesium, resulting in hypomagnesemia [9,15]. Since AKI often occurs without the presence of concurrent hypomagnesemia, and vice versa, it is hypothesized that different mechanisms may be involved in both manifestations of nephrotoxicity. Furthermore, both cis- and carboplatin can cause these forms of nephrotoxicity. Hypomagnesemia is often observed in both cis- and carboplatin-treated patients, whereas AKI is more commonly caused by cisplatin [16,17]. Nephrotoxicity may lead to treatment adaptations, which can affect patient outcomes [18]. 

The identification of risk factors can reduce the risk of nephrotoxicity through closer monitoring and the adoption of more preventive measures. The established clinical risk factors, especially for AKI, are older age, pre-existing renal disease, a history of platinum treatment, high peak concentrations of platinum (resulting from high platinum doses and/or shorter administration times), and the use of other nephrotoxic drugs (e.g., other cytostatic agents and aminoglycosides) [19,20,21]. The effects of genetic variation on, for example, platinum pharmacology, or the sensitivity of the targeted tissues, such as renal tubule cells, may also influence a patient’s likelihood of developing nephrotoxicity during platinum treatment. The pharmacogenetic studies conducted so far have mainly focused on investigating the associations of variants in selected candidate genes with cisplatin-induced AKI. The most heavily investigated genes have been the DNA repair genes, *ERCC1* and *ERCC2*, and the transporter gene, *SLC22A2*, but the reported association between variants and cisplatin-induced AKI could often not be replicated [22,23,24,25,26,27,28,29,30,31,32,33,34,35,36,37,38,39,40]. One recently published genome-wide association study (GWAS), investigating the associations between cisplatin-induced AKI and genetic variants across the whole genome, identified five novel variants in a cohort of adult cancer patients, including a variant in *BACH2* that was also statistically significantly associated in a second validation cohort [41]. For platinum-induced hypomagnesemia, no studies investigating genetic risk factors have been performed to date.

The aim of this study was to clinically and genetically explore platinum-induced nephrotoxicity in a cohort of pediatric brain tumor and adult head–neck cancer patients, including both cisplatin- and carboplatin-treated patients. Genome-wide association analyses were performed with the aim of identifying the genetic risk factors for nephrotoxicity by investigating both acute kidney injury and hypomagnesemia. These data were also used to replicate earlier reported genetic associations. Genetic risk factors have the potential to offer insights into the biological mechanisms involved in the development of toxicities, and could ultimately be used for risk stratification to prevent toxicities from occurring. 

## 2. Materials and Methods

### 2.1. Patients

The study included patients from two cohorts. The first cohort consisted of pediatric brain tumor patients on treatment regimens containing cisplatin and/or carboplatin, treated between 2000 and 2016 at the Radboud University medical center in Nijmegen, the Netherlands, and at the Fondazione IRCCS Istituto Nazionale Tumori in Milan, Italy. Of The patient’ clinical data were retrospectively collected from their medical files. The second cohort included cisplatin-treated adult head–neck tumor patients treated between 2013 and 2017 at the Radboud University medical center in Nijmegen, and the Erasmus University Medical Center, Rotterdam, the Netherlands, who participated in the prospective PRONE study [42]. The current study was approved by local ethical committees. Written informed consent was obtained from the patients and/or their parents/legal guardians.

Patients were eligible for inclusion if: (1) they received primary chemotherapeutic treatment, including a platinum agent, either cisplatin or carboplatin. Treatment regimens depended on tumor type and local treatment protocols, and may or may not have included other chemotherapeutic agents and/or radiation. (2) Patients’ laboratory values were available before and during platinum treatment with serum creatinine and/or magnesium. (3) Patients’ demographic and treatment data were well documented and available, including details on platinum treatment regimens, supportive care, and co-medication. (4) Patients’ stored saliva were available, or they were willing to donate saliva for genome-wide genotyping. Exclusion criteria were as follows: patients were not platinum-naïve, had pre-existent renal disease, or abnormal (age-corrected) baseline values of serum creatinine and serum magnesium (i.e., outside laboratory specific normal ranges) within 4 weeks of the start of platinum treatment. 

### 2.2. Nephrotoxicity

#### 2.2.1. Acute Kidney Injury

Serum creatinine values were used to capture the phenotype acute kidney injury after platinum administration, resulting in a decrease in glomerular filtration rate and, consequently, an increase in serum creatinine. Laboratory results were collected from medical records during platinum treatment and at follow-up. For validation purposes, phenotypes were defined in the manner recently reported by the GWAS of Zazuli et al. [41], in which the serum creatinine values were treated as both continuous and categorical variables. For the continuous variable, the estimated glomerular filtration rate (eGFR) was calculated, both at baseline and at the point at which the highest serum creatinine values were achieved during platinum treatment. For the adults, the CKD-EPI equation, which uses serum creatinine, sex, age, and ethnicity, was applied [43]. Since this equation is not suitable for children, the eGFR of patients aged <18 years was calculated according to the Schwartz formula, which uses serum creatinine and length [44]. To capture decline in eGFR during platinum treatment, a ratio was calculated (worst eGFR/baseline eGFR), in which lower values represent greater toxicity. This ratio was used as continuous phenotype in further analyses. For the categorical variable, the highest serum creatinine value was used to grade the patients according to CTCAE v4.03, ‘Acute kidney injury’, with grade 0 indicating no AKI (‘controls’), and grade 1 or higher indicating AKI (‘cases’) (Table S1).

#### 2.2.2. Hypomagnesemia

To capture impact of platinum treatment on renal tubules, data were collected regarding serum magnesium levels and need for oral or intravenous magnesium supplementation due to renal loss. These data were used to define two phenotypes, which were continuous and categorical. For the continuous phenotype, the lowest measured serum magnesium level during and after platinum treatment was used. For the categorical phenotype, first, the lowest magnesium level during or after treatment was used to grade patients according to CTCAE v4.03 ‘Hypomagnesemia’ (Table S1). Patients with grade 1 or higher were considered cases. Patients with CTCAE grade 0 were assigned as controls if they did not receive magnesium supplementation due to renal loss during treatment. In case of reported therapeutic magnesium supplementation during or after treatment, patients were assigned as cases.

### 2.3. Genotyping

Germline DNA was extracted from saliva (collected using GeneFiX DNA Saliva Collector GFX-02, Isohelix, UK), and isolated using ChemagicStar (Hamilton Robotics, Reno, NV, USA) and the Chemagic STAR DNA Saliva 4k Kit, according to the manufacturer’s protocol. Samples were genotyped with genome-wide coverage using the Illumina Infinium Global Screening Array-24, version 2.0 and version 3.0, performed by Human Genomics Facility at Erasmus MC, Rotterdam, the Netherlands.

Quality control (QC) and imputation were performed in a larger cohort of 848 cancer patients, from which the subset of patients included in this study was extracted. QC started with exclusion of samples with individual call rates below 90%. On the marker level, genetic variants were removed if they showed a call rate below 98%, when minor allele frequency (MAF) was below 0.5%, or when they deviated from the Hardy Weinberg equilibrium (HWE), with a *p*-value below 1 × 10^−6^. To retain a homogenous cohort, principal components (PC) were plotted, excluding individuals considered to be outliers according to PCs 1 and 2. Samples were excluded if they showed sex discrepancies between genotyping and phenotyping data, and in case of relatedness among samples within a cohort (proportion inherited by descent (PI-HAT) above 0.2). Genotyping data were phased and imputed using Eagle (v2.3.5) and Minimac3 software, respectively, with the 1000 Genomes European dataset as a reference panel [45,46]. Imputed variants with an info-score below 0.6 or a MAF below 0.5% were excluded. All steps in the process of phasing, imputation, and QC were performed using the Rapid Imputation and Computational Pipeline for Genome-Wide Association Studies [47]. For genomic location, GRCh37/hg19 was used.

### 2.4. Statistical Analyses

#### 2.4.1. Clinical Characteristics

Potential associations between clinical (patient or treatment) characteristics and continuous nephrotoxicity phenotypes were analyzed using Fisher’s exact, Pearson’s chi-square, Mann–Whitney U, or independent-sample T tests, depending on the type of data and normal distribution, using SPSS Statistics (version 25.0, IBM Corp., Armonk, NY, USA). These were performed separately for creatinine-based and magnesium-based phenotypes. The correlation between decreased eGFR and hypomagnesemia was assessed using Spearman’s rho, Pearson, or point-biserial correlation tests (depending on data type and Gaussian distribution). A two-sided *p*-value of less than 0.05 was considered statistically significant.

#### 2.4.2. Genome-Wide Association Analyses

Based on cohort size, case–control distribution, *p*-value threshold, and genetic model, the predicted power to identify associations with certain effect sizes and allele frequencies was calculated using Quanto (version 1.2.4, Los Angeles, CA, USA). In total, four genome-wide association studies (GWAS) were performed, using the categorical and continuous outcome variables for both the creatinine-based acute kidney injury phenotype and hypomagnesemia phenotype. Linear regression was used for continuous variables, and logistic regression for binary phenotypes (with controls coded as ‘1′, and cases as ‘2′). The GWASs were performed under the assumption of an additive genetic model, using PLINK software (v2.0, Cambridge, MA, USA) [48]. Eigenvalues were used to determine the number of principal components to include as covariates to account for potential population stratification bias. In primary analyses, clinical variables that were statistically significant and associated with the respective phenotype were included as covariates (Section 2.4.1., Clinical Characteristics). Secondary GWAS analyses were performed and included the following clinical variables as covariates in the model: sex, age at diagnosis, disease, primary platinum agent, platinum dose per course, cumulative platinum dose, and treatment with other nephrotoxic agents. Results were compared with the primary GWAS results, to evaluate the impact of addition of these clinical variables in the model on genetic associations. The *p*-value threshold for genome-wide statistical significance was set to 5 × 10^−8^, and the threshold for suggestive significance as *p*-value of 10^−5^.

#### 2.4.3. Replication of Candidate Variants

The variants that were reported to be associated to cisplatin-induced AKI more than once, rs316019 in *SLC22A2*, rs11615 and rs3212986 in *ERCC1*, and rs13181 and rs1799793 in *ERCC2*, were included. Furthermore, statistically significantly (*p* < 5 × 10^−8^) and suggestively (*p* < 10^−5^) associated variants from the GWAS by Zazuli et al. (2021) were investigated in this study [41]. This included a total of 244 genetic variants, with 195 variants for the eGFR-coded analysis (including 5 variants that surpassed the genome-wide significance threshold), and 81 variants for CTCAE-graded analysis, with an overlap of 32 variants. Summary statistics from these variants were obtained from the authors and compared to the results of this study’s association analyses, which were extracted from the results of the eGFR-based GWAS analyses. The Bonferroni-corrected *p*-value threshold for statistical significance for validation analyses was set to *p* < 0.0002, based on the total number of investigated genetic variants (*p* < 0.05 divided by 249 variants).

## 3. Results

### 3.1. Patient and Treatment Characteristics

In total, 249 platinum-treated cancer patients, comprising 147 pediatric brain tumor patients and 102 adult head–neck tumor patients, were eligible and provided informed consent for this study. Six patients did not have any nephrotoxicity data (neither creatinine nor magnesium values). For 25 patients, no saliva was available for genotyping. Of the remaining 218 genotyped subjects, 206 samples passed genetic quality control (Section 3.3., Genotyping) and were therefore included in further analyses. For the creatinine-based analyses, six subjects were excluded as they did not have serum creatinine values (including baseline) available. Five subjects were excluded as they had an age <1 year, for which the Schwartz eGFR formula is not validated, resulting in a total of 195 patients for the creatinine-based analyses. For the magnesium-based analyses, 38 patients did not have magnesium plasma levels available, and two patients had abnormal magnesium plasma levels at baseline, leading to a total of 163 patients.

The clinical characteristics are shown in Table 1, where the patients included in the creatinine-based and magnesium-based analyses are presented separately. The median age in the brain tumor cohort was 8 years (range 1–47), and in the head–neck tumor cohort, it was 59 years (range 28–72). The patients from the brain tumor cohort received a higher median cisplatin dose per cycle (75 mg/m²) and cumulative dose (490 mg/m²) compared to the head–neck tumor patients (40 mg/m² and 240 mg/m², respectively). The adult head–neck tumor patients showed statistically significantly less decline in eGFR compared to the pediatric brain tumor patients. The low-grade glioma patients had statistically significantly lower values of magnesium plasma levels compared to those from the other disease groups. The majority of the patients were primarily cisplatin-treated, accounting for 76.4% and 81.0% of the groups for the creatinine-based and magnesium-based analyses, respectively. The cumulative cisplatin dose ranged from 80–900 mg/m², with the dose per cycle ranging from 30–100 mg/m². The patients who received higher cisplatin doses per cycle (and, consequently, received a higher cumulative dose) had statistically significantly more eGFR decline, but did not have lower magnesium plasma levels. The cumulative carboplatin dose ranged from 200–16,047 mg/m², and the dose per cycle ranged from 35–800 mg/m². The patients with higher cumulative doses of carboplatin had statistically significantly lower magnesium plasma levels. The patients received hydration and preventive intravenous magnesium supplementation, and the children also received mannitol, according to protocol. The treatment often contained other potentially nephrotoxic agents, including cyclophosphamide, etoposide, vincristine, and aminoglycosides. The use of one or more nephrotoxic drugs was statistically significantly associated with a greater decline in eGFR, but not with lower magnesium levels. The data regarding the use of NSAIDs could not be collected from the medical records in a reliable way, but use of NSAIDs is contraindicated during chemotherapy. Amifostine and sodium thiosulfate (potentially nephroprotective agents) were not used. 

Statistically significantly associated clinical variables were included as covariates in the genetic association analyses. For the creatinine-based analyses, these variables were age at diagnosis, platinum dose per cycle, and the use of one or more other nephrotoxic drugs. The diagnosis (either medulloblastoma, low-grade glioma, or head–neck tumor) was not included as a covariate, since it would potentially cause overcorrection due to the fact that it was mainly driven by differences in age and differences in the platinum dose. For the magnesium-based analyses, the covariates included diagnosis and platinum cumulative dose. All the GWAS analyses included the first four principal components as covariates. 

### 3.2. Nephrotoxicity

The characteristics of the nephrotoxicity outcomes are shown in Table 2, which depicts the descriptions and frequencies of the different phenotypes in the total cohort, and separates the data of the primarily cisplatin- and carboplatin-treated patients. The median of the worst eGFR in the analyzed cohort was 89.2 mL/min/1.73 m² (range 31.6–179.3), with no difference between the cisplatin- and carboplatin treated patients. The CTCAE-AKI graded phenotype consisted of 26 cases and 169 controls, with most of the cases (19 out of 26) assigned as grade 1. The median lowest magnesium plasma level after platinum treatment was 0.76 mmol/L (range 0.19–0.91), with the carboplatin-treated patients showing more magnesium loss compared to the cisplatin-treated group (medians of 0.74 vs. 0.77, respectively). The CTCAE hypomagnesemia grading resulted in 132 patients with grade 0, 24 patients with grade 1, and 7 patients with grades 2–4. A total of 22 patients (13.5%) received therapeutic magnesium supplementation during or after platinum treatment, of whom the majority (15 out of 22) were graded as grade 1 or higher. The seven remaining patients were assigned as cases solely based on their receival of therapeutic magnesium supplementation, due to further missing data regarding their magnesium plasma levels. Altogether, these outcomes of the case–control designation resulted in a total of 38 cases and 125 controls, with a relatively high number of cases in the carboplatin-treated group (13 patients, 41.9%) compared to the cisplatin-treated group (25 patients, 18.9%). No differences in baseline magnesium plasma levels were observed between the cases and the controls.

The correlation between the creatinine-based and magnesium-based phenotypes was examined in 159 patients, for whom sufficient data for both phenotypes were available (Table S2). No clear correlations were identified between the creatinine- and magnesium-based phenotypes, except a statistically significant correlation between decreased eGFR and CTCAE-coded hypomagnesemia (Pearson correlation coefficient −0.210).

### 3.3. Genotyping

Genome-wide genotyping, QC, and imputation were performed in a larger cohort of 848 cancer patients. This cohort was genotyped for 576,952 variants. The marker QC resulted in the removal of a total of 103,365 variants (103,121 variants due to low MAF or low marker call rates; 244 variants due to HWE deviation). The remaining 473,587 variants were used for the imputation. After the imputation and post-imputation QC, 9,799,032 genetic variants were available for the analyses. The sample QC resulted in the exclusion of 203 subjects (108 subjects with low sample call rates, 91 subjects were excluded as a result of the PCA, as well as 3 subjects with sex discrepancies and 1 subject showing high relatedness scores). Of the 645 subjects remaining in the dataset after the QC, the inclusion and exclusion criteria for this genetic study were met by 195 patients for the creatinine-based analyses, and 163 patients for the magnesium-based analyses.

### 3.4. Creatinine-Based Analyses

#### 3.4.1. GWASs

The genome-wide association analyses for the decline in eGFR (ratio, as a continuous variable) and the CTCAE-AKI phenotype (as a binary variable) were performed, both including 195 patients. The post hoc power calculations for the CTCAE-AKI phenotype showed a power of >80% to detect genetic variants, with an odds ratio of 8 and higher and an allele frequency between 0.20–0.30 at an alpha of 5 × 10^−8^ (Figure S1A). 

**Figure 1:**
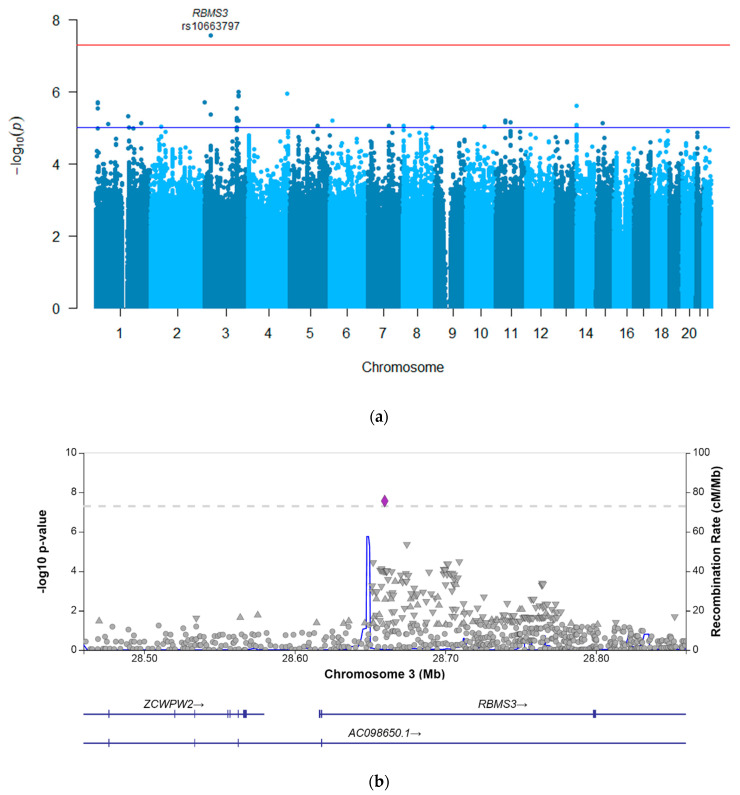
Results of GWAS investigating eGFR decrease after platinum. *p*-values were generated using logistic regression. (**a**) Manhattan plot showing the associations between eGFR decrease and genetic variants (blue dots), plotted against chromosomal position (x-axis) and −log10 *p*-value (y-axis). The red line represents the *p*-value threshold for genome-wide statistical significance (*p* < 5 × 10^−8^). The blue line represents a suggestive *p*-value threshold (*p* < 10^−5^). (**b**) LocusZoom plot showing the variant rs10663797 and its surrounding region, which was the variant most strongly associated with eGFR decrease. This variant (purple diamond) is located in the intronic region of the *RBMS3*. The p-values on the −log10 scale are plotted on the left y-axis. The right y-axis indicates the regional recombination rate (cM/Mb), depicted by the blue line on the plot (where peaks indicate recombination hot spots). The chromosomal position is plotted along the x-axis, along with the genes located in that region.

The results of the GWAS investigating the eGFR decline are presented in Figure 1. In this analysis, since the ratio (worst eGFR/baseline eGFR) was used as the outcome, a coefficient below zero corresponded to an increased risk of eGFR decline when a patient carried the effect allele, and a coefficient above zero suggested a decreased risk. This analysis resulted in one genome-wide significant hit, *RBMS3* rs10663797, which showed a coefficient of −0.10 (95% confidence interval −0.13–−0.06) with a *p*-value of 2.72 × 10^−8^ (Table 3), indicating that patients carrying the AC deletion had statistically significantly greater eGFR decline (i.e., a lower eGFR ratio) after the platinum treatment compared to the patients carrying the AC insertion. This indel variant in the intronic region of *RBMS3* had a minor allele frequency of 0.27 in the study’s cohort. Furthermore, 31 other genetic variants showed a *p*-value of <10^−5^, representing 20 independent loci, for which the summary statistics are provided in Table S3.

For the GWAS using the CTCAE-graded phenotype, an odds ratio above 1 indicates a presumed increased risk for AKI for carriers of the effect allele, and below 1 indicates a decreased risk. The analysis did not result in genome-wide significant hits. A total of 25 variants, representing 4 independent loci, showed a *p*-value of <10^−5^ (Table S4). The most strongly associated variant in the CTCAE-graded GWAS was rs77890968, an intergenic variant on chromosome 15 that showed an odds ratio of 15.9 (95% confidence interval 5.0–50.4) with a *p*-value of 2.55 × 10^−6^, implying an increased risk of AKI for carriers of the effect allele, which was also the case for this variant in the eGFR GWAS (coef. −0.09, 95% CI 0.03–−0.15, *p*-value 0.004). When comparing the results of both the creatinine-based GWASs, the directions of the effects of all the 35 variants with a *p*-value of <10^−5^ from the eGFR GWAS were the same in the CTCAE-graded GWAS (data not shown). Most notably, the result from the genome-wide significant hit from the eGFR GWAS, *RBMS3* rs10663797, also showed a damaging effect of the effect allele (OR 5.69, 95% CI 2.54–12.74, *p*-value 2.33 × 10^−5^) in the CTCAE-graded analysis (Table 3).

The secondary analyses, including all the clinical variables as covariates, did not affect the most strongly associated variants of either GWAS, with the variant *RBMS3* rs10663797 remaining significant genome-wide (coef. −0.09 (95% CI −0.13–−0.06, *p*-value 5.79 × 10^−8^) in the eGFR analysis. The suggestively associated variants (*p*-value < 10^−5^) showed a large overlap across the secondary and primary analyses, suggesting a limited effect of the included clinical variables on the genetic associations (data not shown).

#### 3.4.2. Replication of Previously Reported Associations

A comparison of the statistically significantly associated variants in the study by Zazuli et al. with this study’s results is depicted in Table 4. Both studies analyzed eGFR decline. Two of five variants were excluded from this study’s QC, and could therefore not be analyzed. The other three variants, rs199659233 and rs556958738 in *ARPC1A* and rs4388268 in *BACH2*, did not show an association with eGFR decline in this study’s cohort, with *p*-values of 0.899, 0.899, and 0.443, respectively. The directions of the effect sizes were in line with the discovery study, showing a decreased risk of eGFR decline for carriers of the T-allele of the *ARPC1A* variants, and an increased risk for the A-allele carriers of the variant in *BACH2*. 

The suggestively associated (*p*-value < 10^−5^) variants in the analysis by Zazuli et al. were explored in this study’s results. Of the 190 genetic variants suggestively associated with eGFR decline after the platinum treatment, the results of 181 variants were available. None of these variants showed a statically significant association (Bonferroni-corrected *p*-value < 0.0002) in this study’s cohort. Variant rs17725981 showed the strongest association (*p* = 0.019), but the direction of the effect contrasted with the findings of the discovery study. In total, 70 out of the 181 genetic variants showed the same direction of effect in both studies. When comparing the results of the CTCAE-AKI-graded analyses of both studies, 79 out of 85 variants were available, of which 33 variants showed the same direction of effect in both studies. None of the variants showed a statistically significant association with AKI. The variants rs11608760 and rs61276435 were most strongly associated (*p* = 0.04 for both variants) and showed the same direction of effect as the discovery study’s results.

The analyses of the five previously reported candidate variants in the genes *SLC22A2*, *ERCC1* and *ERCC2* did not show a statistically significant association with eGFR decline or CTCAE-AKI in this study’s cohort (Table S7).

### 3.5. Magnesium-Based GWASs

Two genome-wide association analyses were performed using magnesium-based phenotypes in 163 patients. One analysis used the lowest magnesium plasma level during treatment (as a continuous variable), and the other analysis used these data graded according to CTCAE in combination with the need for magnesium supplementation (as a binary variable). For this outcome, post hoc power calculations using the binary phenotype showed that this study’s power to detect genetic variants with an odds ratio of 8 and an allele frequency of 0.20 was 70% (Figure S1B).

**Figure 2:**
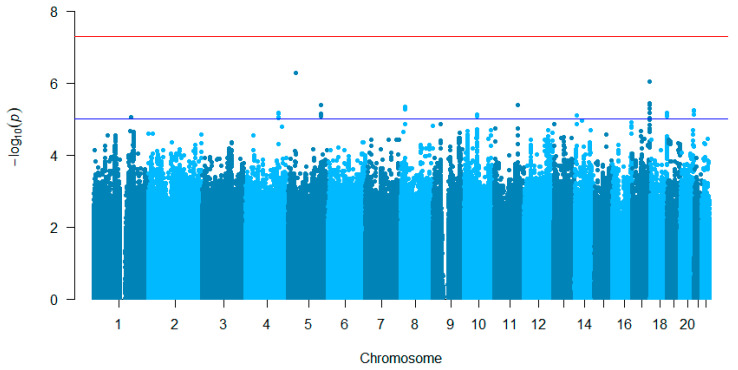
Results of GWAS investigating lowest magnesium plasma level after platinum treatment. The *p*-values were generated using logistic regression. The Manhattan plot shows the associations between lowest magnesium plasma level and genetic variants (blue dots), plotted against chromosomal position (x-axis) and -log10 *p*-value (y-axis). The red line represents the *p*-value threshold for genome-wide statistical significance after Bonferroni correction for multiple testing (*p* < 5 × 10^−8^), with no genetic variants surpassing this threshold. The blue line represents a suggestive *p*-value threshold (*p* < 10^−5^), with 35 hits surpassing this threshold (representing 11 independent loci). The top hit is the genetic variant, *RAI4* rs563097889, on chromosome 5 (*p =* 5.16 × 10^−7^).

The GWAS for the continuous hypomagnesemia phenotype resulted in no genetic variants surpassing the *p*-value threshold for genome-wide significance (Figure 2). A total of 35 genetic variants showed a *p*-value of less than 10^−5^, representing 11 independent loci (Table S5). The strongest associated variant was the indel variant, rs563097889, in the intronic region of gene *RAI4* on chromosome 5 (coef. 0.11, 95% CI 0.02–0.06, *p* = 9.02 × 10^−6^). The GWAS for the dichotomized hypomagnesemia phenotype resulted in one suggestively associated variant (rs6496125, *p* = 7.54 × 10^−6^, Table S6).

A literature search yielded no results regarding genetic association studies investigating platinum-induced tubular magnesium loss (or hypomagnesemia). 

## 4. Discussion

Nephrotoxicity is one of the dose-limiting side effects of platinum agents. This study investigated novel and previously described genetic risk factors for platinum-induced nephrotoxicity, including AKI and hypomagnesemia. The study identified a novel association between the genetic variant rs10663797 in *RBMS3* and AKI. The patients who carried the two base-pair deletions had statistically significantly greater eGFR decline than the patients who carried the insertion at this locus. The previously identified genetic variants from candidate-gene and genome-wide association studies investigating AKI were not validated at multiple-testing-adjusted levels of significance in this study’s cohort. No genetic risk loci could be identified for platinum-induced hypomagnesemia. 

*RBMS3*, RNA binding motif single-stranded interacting protein 3, encodes for a RNA-binding protein, which is involved in gene transcription, cell-cycle progression, and apoptosis [49]. The identified variant, rs10663797, located in the intronic region of *RBMS3*, was not previously identified in the GWASs of the other traits. The other variants in *RBMS3* showed associations with, inter alia, blood pressure, lung function, and bone mineral density, but no studies described a clear association with kidney-related traits [50]. *RBMS3* is most highly expressed in the arteries and muscles, with low expression in the kidney cortex [51], and is not known from the literature to be involved in platinum pathways or renal disease. This gene was previously described in association with intellectual disability and Heimler syndrome, but these patients did not have phenotypes with renal impairment [52,53]. Studies have reported that higher *RBMS3* expression in tumor cells is associated with more favorable disease outcomes in, inter alia, esophageal, nasopharyngeal, lung, and gastric cancer, which are tumor types that are often platinum-treated [54,55,56]. An additional study implied that the loss of *RBMS3* resulted in the development of platinum resistance in ovarian tumor cells [57]. One could hypothesize that higher *RBMS3* expression eventually leads to the increased apoptosis of platinum-exposed tumor cells, and, in the same way, that it might lead to the increased apoptosis of platinum-exposed cells in the kidneys. However, since a deletion, such as *RBMS3* rs10663797, usually does not lead to increased gene expression, the exact nature of the identified associations remains unclear. 

This study was unable to replicate recently published GWAS findings by Zazuli et al. [41]. Most notably, the study’s main finding, an association between a genetic variant in *BACH2* and the cisplatin-induced decrease in eGFR in adult cancer cohorts, was not present in this study’s cohort, although the direction of the effect was the same in both studies. This was also the case for two other variants reported to be statistically significantly associated in the discovery study. Cohort differences regarding disease types, clinical risk factors, and treatment regimens could have played a role in the discrepancies between the study outcomes, but since these are largely corrected for in statistical analyses, robust true associations should be detected despite these differences. Another reason for the discrepancy in the results could be that the current study was insufficiently powered to detect the variants, leading to false-negative findings. However, the effect size reported by the discovery study was large (coefficient of −8.4 in an analysis of 608 patients), and no trend towards association was observed in this study (unadjusted *p*-value of 0.443). Furthermore, the association could have been a false-positive finding in the discovery study, but this is unlikely, given that the association was validated in an adult cohort. Since this study is the first to report on the reproducibility of these recent findings, additional studies investigating these associations in independent cohorts will shed light on the true nature of the role of *BACH2*, and others, in platinum-induced nephrotoxicity. 

No genetic risk factors could be established for platinum-induced hypomagnesemia. To the authors’ knowledge, this study is the first to perform a genetic association investigating this trait. A stop-gain variant in *ENDOV* approached the genome-wide significance threshold (*p* = 3.74 × 10^−6^, Table S5), and is predicted to be potentially damaging to the protein [58]. *ENDOV* is located intercellularly, and is highly expressed in many tissues, including those of the kidneys [51,59]. Interestingly, one of its molecular functions is magnesium ion binding; however, this is mostly described in relation to DNA repair and not necessarily to circulating plasma magnesium [60,61]. This study provides too little evidence to propose this as a candidate risk gene, but it could generate further interest should (larger) future studies identify a role for this gene in platinum-induced hypomagnesemia. 

Both cisplatin and carboplatin were analyzed in this study. Many studies in the past investigated the nephrotoxic potency of both agents, with carboplatin typically described as less nephrotoxic than cisplatin [62]. Unlike cisplatin, carboplatin is not transported by OCT-2 within proximal tubule cells, resulting in lower concentrations of platinum within the cells and, therefore, less damage [63,64]. However, both agents still have the potential to cause AKI and hypomagnesemia via the same biological pathways; combining these cohorts in genetic association analyses increases the cohort size and should not lead to the loss of potential signals. In this study’s cohort, both AKI and hypomagnesemia occurred in both the cisplatin- and the carboplatin-treated group. Although not statistically significantly different, hypomagnesemia was more common in the carboplatin-treated patients, and AKI occurred more frequently in the cisplatin-treated patients, in concordance with the results of most previous studies [16,65]. Higher doses per course, and, therefore, higher peak plasma concentrations, were statistically significantly associated with greater decreases in eGFR for the cisplatin-treated patients in this study, which is an established risk factor [66]. For carboplatin and hypomagnesemia, rather than the dose per course, a higher cumulative carboplatin dose was shown to be statistically significantly associated with lower magnesium plasma levels. Although this study was not designed to address this, this observation could suggest that frequent carboplatin administration and, therefore, high cumulative doses, put patients at risk for the development of hypomagnesemia.

This study has its limitations. Firstly, both AKI and hypomagnesemia are clinical traits that are influenced by a multitude of clinical factors. Sufficient and reliable data were available for many important factors, such as platinum dosing regimens, patient demographics, and nephrotoxic co-medication. However, the retrospective character of this study inevitably led to missing variables. Clinical factors, such as malnutrition, cardiovascular status, hypoalbuminemia, and detailed information on diuretics (such as the dose and frequency) were not sufficiently documented and could therefore not be collected and analyzed in a reliable way [67,68]. Malnutrition in particular is very common in cancer patients due to treatment-induced nausea and mucositis, which can influence the balance of electrolytes, including magnesium. Furthermore, excessive weight loss and reduced muscle mass due to malnutrition impact creatinine values. Future (prospective) studies should take these clinical factors into account. Secondly, the majority of the included patients were on multi-agent chemotherapeutic regimens containing agents that could potentially affect the kidneys as well. Despite this study’s effort to map these other agents and include them in the statistical analyses, one cannot rule out that these other agents might have affected the nephrotoxicity phenotypes as well, thereby clouding associations that were specific to platinum. Thirdly, due to the relatively small cohort size for genome-wide analyses in this study, only genetic variants with relatively high effect sizes could be detected, which was demonstrated in the power analyses. Genetic variants with smaller effect sizes could still have a large impact on the outcomes, if combined in a pharmacogenetic profile or in the identification novel therapeutic targets. Thus, large (international) studies remain important in order to pinpoint which genes and variants play a role in the interpatient variability in platinum-induced nephrotoxicity. 

The aim of this study was to genetically explore platinum-induced nephrotoxicity. The genome-wide analyses resulted in a novel association between an indel variant in *RBMS3* and platinum-induced AKI. This gene was not previously described in relation to kidney-related traits, but it was shown to play a role in the progression of platinum-treated tumors. The replication and validation of this finding is necessary to confirm whether this is a true association, with functional studies supporting the finding that this gene is involved in the development of platinum-induced nephrotoxicity. Ultimately, this may contribute to a better understanding of the interpatient variability in platinum-induced nephrotoxicity, which can serve as an entry point for preventive measures to protect patients against these side effects.

## Figures and Tables

**Table 1:** Characteristics of patient population.

		Creatinine (*n* = 195)			Magnesium (*n* = 163)		
		*n* ^a^	*n* (%), or Median (Range) ^b^	*p* ^b^	*n* ^a^	*n* (%), or Median (Range) ^b^	*p* ^b^
***Demographics***							
Male sex		195	120 (61.5%)	0.728	163	107 (65.6%)	0.439
Age at diagnosis (in years)		195	21 (1–72)	**0.030**	163	48.0 (0–72)	0.451
	<18 years old	195	90 (46.2%)	**0.002**	163	69 (42.3%)	0.284
	≥18 years old		105 (53.8%)			94 (57.7%)	
Self-reported Caucasian ethnicity		195	195 (100.0%)	-	163	163 (100.0%)	-
Diabetes mellitus		195	1 (0.5%)	0.400	163	1 (0.6%)	0.768
***Disease and treatment***							
Diagnosis							
	Medulloblastoma	195	72 (36.9%)	**0.012**	163	41 (25.2%)	**0.004**
	Low-grade glioma		34 (17.4%)			36 (22.1%)	
	Head–neck tumor		89 (45.6%)			86 (52.8%)	
Received radiotherapy		195	161 (82.6%)	0.085	163	129 (79.1%)	0.247
Primary platinum agent							
	Cisplatin	195	149 (76.4%)	0.236	163	132 (81.0%)	0.170
	Carboplatin		46 (23.6%)			31 (19.0%)	
Cisplatin treatment							
	Cisplatin cumulative dose (mg/m²)	150	240 (80–900)	**0.010**	133	240 (80–900)	0.998
	Cisplatin dose per cycle (mg/m²)	150	70 (30–100)	**9 × 10^−10^**	133	70 (30–100)	0.495
Carboplatin treatment							
	Carboplatin cumulative dose (mg/m²)	63	800 (200–16,047)	0.170	39	1600 (200–16,047)	**0.0001**
	Carboplatin dose per cycle (mg/m²)	62	550 (35–800)	0.755	38	550 (35–800)	0.131
Hydration per protocol (in L/m²/cycle)		191	2.5 (0.5–5.3)	0.137	161	2.5 (0.8–5.3)	0.189
***Use of diuretics***							
Furosemide		179	42 (23.5%)	0.277	111	41 (36.9%)	0.903
***Use of potentially nephrotoxic drugs***							
Use of one or more nephrotoxic drugs		195	106 (54.4%)	**0.003**	163	77 (47.2%)	0.422
Cyclophosphamide		195	47 (24.1%)		163	29 (17.8%)	
Etoposide		195	55 (28.2%)		163	40 (24.5%)	
Vincristine		195	84 (43.1%)		163	56 (34.4%)	
Methotrexate		195	30 (15.4%)		163	15 (9.2%)	
Aminoglycosides		193	17 (8.8%)		161	7 (4.3%)	

*n*, number of patients; *p*, *p*-value. ^a^ Number of patients with data available for this variable. ^b^ Results of covariate analysis with eGFR decline and lowest magnesium level, where the choice of statistical test (Pearson correlation, Spearman’s rho, Mann–Whitney U, or independent sample *t*-test) depended on data type and Gaussian distribution. Two-sided *p*-value threshold for significance: 0.05. Clinical variables were tested for association with decrease in eGFR (ratio) and lowest serum magnesium level.

**Table 2:** Characteristics of nephrotoxicity outcomes.

Creatinine-Based Analyses					
			Total	Cisplatin-Treated	Carboplatin-Treated
			*n* = 195	*n* = 149	*n* = 46
***Continuous***					
Worst eGFR, in mL/min/1.73 m² (median, range)			89.2 (31.6–179.3)	89.1 (31.6–138.6)	90.1 (56.6–179.3)
Ratio eGFR, worst/baseline (median, range)			0.9 (0.3–1.3)	0.9 (0.3–1.3)	0.9 (0.4–1.2)
***Binary***					
CTCAE v4.03 ‘Acute kidney injury’					
	Controls: grade 0 (*n*, %)		169 (86.7%)	129 (86.6%)	40 (87.0%)
	Cases: grade 1 or higher (*n*, %)		26 (13.3%)	20 (13.4%)	6 (13.0%)
		Grade 1 (*n*)	19	14	5
		Grade 2 (*n*)	6	5	1
		Grade 3 (*n*)	1	1	0
		Grade 4 (*n*)	0	0	0
		Grade 5 (*n*)	0	0	0
**Magnesium-based analyses**					
			**Total**	**Cisplatin-treated**	**Carboplatin-treated**
			*n* = 163	*n* = 132	*n* = 31
***Continuous***					
Lowest magnesium plasma level, in mmol/L(median, range)			0.76 (0.19–0.91)	0.77 (0.48–0.91)	0.74 (0.19–0.87)
***Binary***					
Controls: CTCAE grade 0, AND no therapeutic magnesium supplementation (*n*, %)			125 (76.7%)	107 (81.1%)	18 (58.1%)
Cases: CTCAE grade 1 or higher, OR therapeutic magnesium supplementation (*n*, %)			38 (23.3%)	25 (18.9%)	13 (41.9%)
CTCAE v4.03 ‘Hypomagnesemia’					
	Grade 0 (*n*)		132	113	19
	Grade 1 (*n*)		24	18	6
	Grade 2 (*n*)		4	1	3
	Grade 3 (*n*)		1	0	1
	Grade 4 (*n*)		2	0	2
	Grade 5 (*n*)		0	0	0
Received therapeutic magnesium supplementation (*n*, %)			22 (13.5%)	10 (9.3%)	12 (38.7%)

*n*, number of patients; eGFR, estimated glomerular filtration rate; CTCAE, common terminology criteria for adverse events.

**Table 3:** Results of genome-wide significant variant from creatinine-based GWAS.

								eGFR (Continuous)			CTCAE-AKI (Binary)		
Variant	Position ^a^	Chr	Gene	Variant Type	Effect Allele	Non-Effect Allele	MAF	Coef. ^b^	95% CI	*p*	OR ^c^	95% CI	*p*
rs10663797	28,659,744	3	*RBMS3*	intronic	delAC	insAC	0.27	−0.10	−0.13–−0.06	2.72 × 10^−8^	5.69	2.54–12.74	2.33 × 10^−5^

Chr, chromosome; MAF, minor allele frequency; Coef., coefficient; CI, confidence interval; *p*, uncorrected *p*-value; OR, odds ratio. ^a^ Location on genome build GRCh37/hg19. ^b^ Coefficient below zero represents an increased risk of eGFR reduction when carrying the tested allele, and above zero indicates a decreased risk. ^c^ Odds ratio above 1 indicates an increased risk of eGFR reduction when carrying the tested allele, and below 1 indicates a decreased risk.

**Table 4:** Replication results of variants found to be statistically significantly associated by Zazuli et al. (2021) [41]

Gene	Variant	Data from Discovery Study Zazuli et al. (2021) [41]					Results Current Study (GWAS with eGFR Decline Phenotype)					Comparison			
		Effect Allele	Beta	95% CI		*p*	Effect Allele	Coef	95% CI		*p*	Effect Allele	Non-Effect Allele	Direction Zazuli et al. ^a^	Direction Current Study ^a^
*TMEM225B*	rs17161766	A	−28.91	−38.80	−19.10	7.823 × 10^−9^	NA	NA	NA	NA	NA	+	G	+	NA
*-*	chr7:98951080	CTTAT	−27.19	−36.50	−17.90	9.485 × 10^−9^	NA	NA	NA	NA	NA	+	C	+	NA
*ARPC1A*	rs199659233	T	28.65	18.70	38.60	1.473 × 10^−8^	C	0.008	−0.115	0.130	0.899	-	C	-	-
*ARPC1A*	rs556958738	T	28.65	18.70	38.60	1.473 × 10^−8^	C	0.008	−0.115	0.131	0.899	-	C	-	-
*BACH2*	rs4388268	A	−8.37	−11.40	−5.40	3.845 × 10^−8^	A	0.013	−0.020	0.045	0.443	+	G	+	+

^a^ Direction of effect, where (+) represents an increased risk of eGFR reduction when carrying the effect allele, and (-) a decreased risk. Chr, chromosome; BP, base-pair position on genomic build GRCh37/hg19; MAF, minor allele frequency; Coef., coefficient; 95% CI, 95% confidence interval; *p*, *p*-value; OR, odds ratio; NA, not available.

## Data Availability

Summary statistics for all the analyses will be available at RIS (Research Information Services), the current research information system of Radboud University, Nijmegen, the Netherlands.

## Supplementary Materials

The following supporting information can be downloaded at: https://www.mdpi.com/article/10.3390/jpm12060892/s1. Table S1: NCI Common Terminology Criteria for Adverse Events (CTCAE), version 4.03, used for grading of nephrotoxicity phenotypes. Table S2: Correlation between creatinine-based and magnesium-based phenotypes in 159 patients. Figure S1: Results of power calculations for creatinine-based and magnesium-based analyses. Table S3: 32 genetic variants suggestively associated (*p* < 10^−5^) with decline in eGFR after platinum treatment. Table S4: 25 genetic variants suggestively associated (*p* < 10^−5^) with acute kidney injury (coded according to CTCAE-AKI v4.03) after platinum. Table S5: 35 genetic variants suggestively associated (*p* < 10^−5^) with lowest magnesium plasma level after platinum treatment. Table S6: One genetic variant suggestively associated (*p* < 10^−5^) with hypomagnesemia (coded according to CTCAE-AKI v4.03 and need for magnesium supplementation) after platinum treatment. Table S7: Results of genetic association analyses of five candidate variants.
