# Supplementary material for: Genome-Wide Analyses of Nephrotoxicity in Platinum-Treated Cancer Patients Identify Association with Genetic Variant in RBMS3 and Acute Kidney Injury
Source: J Pers Med. 2022 May 28;12(6):892. doi: 10.3390/jpm12060892 (PMC9224783; doi:10.3390/jpm12060892)
Supplement: Supplementary file 1 [file jpm-12-00892-s001.zip › jpm-1716852-supplementary.pdf]

SUPPLEMENTARY MATERIALS

Table S1: NCI Common Terminology Criteria for Adverse Events (CTCAE) version 4.03 used for grading of nephrotoxicity phenotypes

**Renal and urinary disorders / Metabolism and nutrition disorders**						
**Adverse event**	**Grade 0**	**Grade 1**	**Grade 2**	**Grade 3**	**Grade 4**	**Grade 5**
**Acute kidney injury**	Creatinine level increase of <0.3 mg/dL; creatinine <1.5 x above baseline	Creatinine level increase of >0.3 mg/dL; creatinine 1.5 - 2.0 x above baseline	Creatinine 2.0 – 33.0 x above baseline	Creatinine >3.0 x baseline or >4.0 mg/dL; hospitalization indicated	Life-threatening consequences; dialysis indicated	Death
Definition: A disorder characterized by the acute loss of renal function and is traditionally classified as pre-renal (low blood flow into kidney), renal (kidney damage) and postrenal causes (ureteral or bladder outflow obstruction).						
**Hypomagnesemia**	>LLN	<LLN - 1.2 mg/dL; <LLN - 0.5 mmol/L	<1.2 - 0.9 mg/dL; <0.5 - 0.4 mmol/L	<0.9 - 0.7 mg/dL; <0.4 - 0.3 mmol/L	<0.7 mg/dL; <0.3 mmol/L; life-threatening consequences	Death
Definition: A disorder characterized by laboratory test results that indicate a low concentration of magnesium in the blood						

Table S2: Correlation between creatinine-based and magnesium-based phenotypes in 159 patients

	**Decrease in eGFR**	**CTCAE Acute kidney injury**	**Lowest magnesium plasma level**	**CTCAE Hypomagnesemia + suppletion**
**Decrease in eGFR**	1.000			
**CTCAE Acute kidney injury**	-0.730**	1.000		
**Lowest magnesium plasma level**	0.050	-0.031	1.000	
**CTCAE Hypomagnesemia + suppletion**	-0.210**	0.084	-0.673 **	1.000

** Correlation is statistically significant (two-tailed *p*-value <0.05)

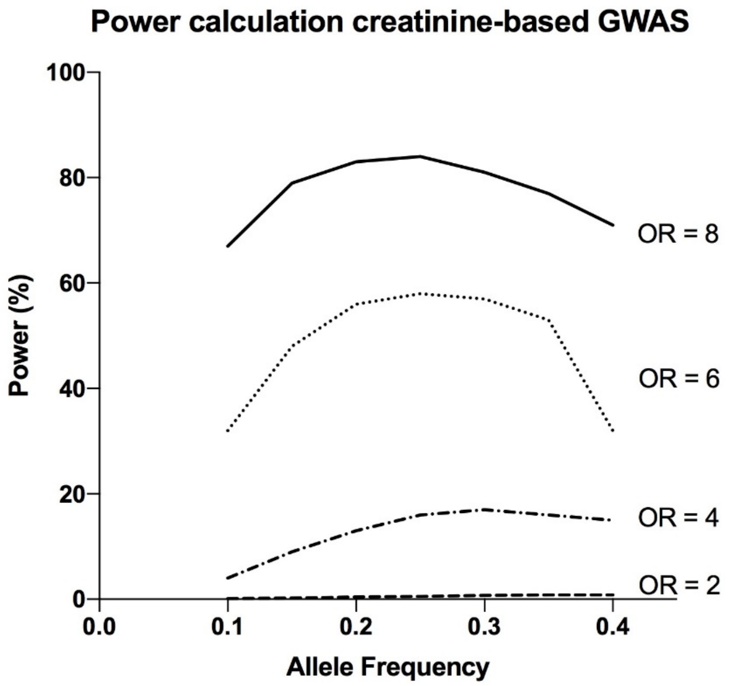

(A)

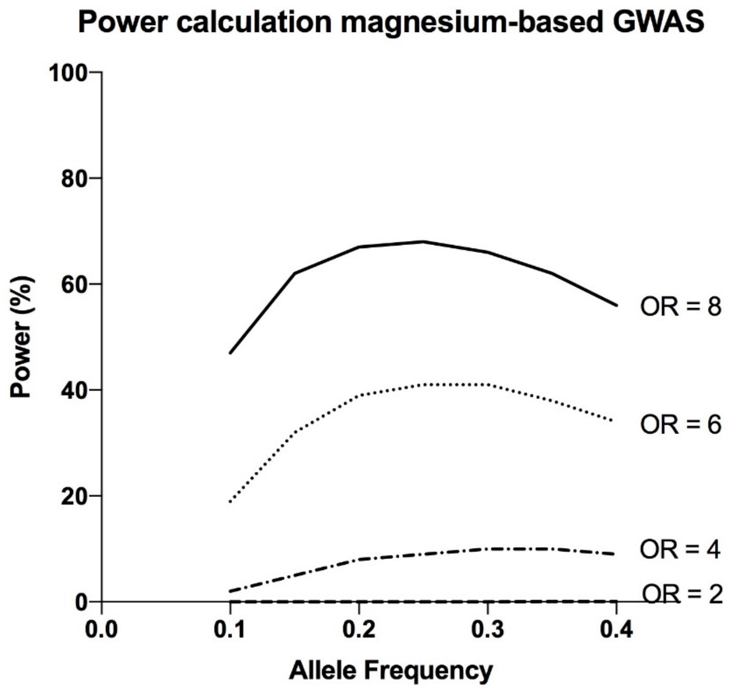

(B)

Figure S1: Results of power calculations for **(A)** creatinine-based analyses (n=195) and **(B)** magnesium-based analyses (n=163). In both calculations, binary phenotypes were used to calculate predicted power. Calculations were performed using Quanto (version 1.2.4, Los Angeles, CA).

Table S3: 32 genetic variants suggestively associated ($p < 10^{-5}$) with decline in eGFR after platinum treatment

rsID	Chr	Position [1]	Nearest gene	Consequence	Effect allele	Non-effect allele	Coef [2]	95 CI (lower)	95 CI (upper)	P
rs10663797	3	28,659,744	*RBMS3*	Intronic	D	I	-0.10	-0.13	-0.06	2.72E-08
rs193202648	3	153,825,916	*ARHGEF26-AS1*	Intronic	T	C	-0.20	-0.28	-0.12	1.01E-06
rs62333844	4	179,936,333	*LINC01098*	Intergenic	A	G	0.34	0.21	0.47	1.13E-06
rs79586953	3	153,916,667	*ARHGEF26*	Intronic	C	A	-0.16	-0.22	-0.10	1.28E-06
rs61791503	3	153,745,224	*ARHGEF26-AS1*	Intronic	C	T	-0.16	-0.22	-0.10	1.32E-06
rs4684362	3	2,882,301	*CNTN4*	Intronic	G	T	-0.13	-0.19	-0.08	1.93E-06
rs71641052	1	8,628,362	*RERE*	Intronic	C	T	-0.09	-0.13	-0.05	2.02E-06
rs17386448	1	8,646,318	*RERE*	Intronic	C	T	-0.09	-0.13	-0.05	2.15E-06
rs57200240	14	21,314,704	*RNASE1*	Intergenic	T	C	-0.07	-0.10	-0.04	2.45E-06
rs12753546	1	8,548,509	*RERE*	Intronic	C	T	-0.09	-0.12	-0.05	2.90E-06
rs5853431	3	149,368,444	*WWTR1*	Intronic	D	I	0.07	0.04	0.10	2.96E-06
rs4539902	3	28,674,463	*RBMS3*	Intronic	A	C	-0.07	-0.10	-0.04	4.37E-06
rs143103033	1	146,999,990	*LINC00624*	Intergenic	A	G	0.37	0.21	0.52	4.84E-06
rs6779455	3	149,364,473	*WWTR1*	Intronic	C	T	0.07	0.04	0.09	5.40E-06
rs11751243	6	12,899,336	*PHACTR1*	Intronic	A	G	-0.22	-0.31	-0.13	6.28E-06
-	3	153,704,751	*ARHGEF26-AS1*	Intergenic	T	C	-0.15	-0.21	-0.09	6.39E-06
rs10935769	3	149,367,044	*WWTR1*	Intronic	T	G	0.07	0.04	0.09	6.39E-06
rs117755171	11	43,751,590	*HSD17B12*	Intronic	C	G	-0.23	-0.33	-0.13	6.40E-06
rs563240554	11	64,750,273	*BATF2*	Intergenic	I	D	0.14	0.08	0.20	7.11E-06
rs150151732	11	43,836,832	*HSD17B12*	Intronic	C	T	-0.24	-0.35	-0.14	7.18E-06
rs530299513	1	204,814,423	*NFASC*	Intronic	D	I	0.08	0.04	0.11	7.33E-06
rs56377033	15	49,209,612	*SHC4*	Intronic	A	G	-0.30	-0.43	-0.18	7.64E-06
rs10493209	1	56,841,669	*PLPP3*	Intergenic	G	A	0.25	0.14	0.35	8.09E-06
rs12884091	14	21,314,696	*RNASE1*	Intergenic	A	C	-0.07	-0.09	-0.04	8.59E-06
rs11770742	7	97,242,501	*TAC1*	Intergenic	C	T	0.15	0.09	0.22	8.63E-06
rs12882414	14	21,314,697	*RNASE1*	Intergenic	G	T	-0.07	-0.09	-0.04	8.67E-06
rs549475905	5	126,380,462	*C5orf63*	Non-coding transcript variant	C	T	0.19	0.11	0.27	8.73E-06
rs13274501	8	5,412,596	*CSMD1*	Intergenic	T	C	-0.08	-0.12	-0.05	8.80E-06
rs12882422	14	21,314,705	*RNASE1*	Intergenic	G	A	-0.07	-0.09	-0.04	8.83E-06
rs142336622	10	84,456,124	*NRG3*	Intronic	C	A	0.26	0.15	0.37	9.19E-06
rs11681101	2	48,983,817	*STON1-GTF2A1L*	Intronic	C	T	-0.07	-0.10	-0.04	9.59E-06
rs7616812	3	149,365,500	*WWTR1*	Intronic	C	T	-0.07	-0.10	-0.04	9.71E-06

rsID, variant identifier (dbSNP); chr, chromosome; coef, coefficient; CI, confidence interval; P, uncorrected *p*-value

[1] Location on genome build GRCh37/hg19

[2] Coefficient below zero represents an increased risk of eGFR reduction when carrying the tested allele, and above zero a decreased risk

Table S4: 25 genetic variants suggestively associated ($p < 10^{-5}$) with acute kidney injury (coded according to CTCAE-AKI v4.03) after platinum

rsID	Chr	Position [1]	Nearest gene	Consequence	Effect allele	Non-effect allele	OR [2]	95 CI (lower)	95 CI (upper)	P
rs77890968	15	98,820,481	*FAM169B*	Intergenic	T	C	15.92	5.02	50.42	2.55E-06
rs56007891	5	91,325,250	*ARRDC3*	Intergenic	I	D	8.64	3.43	21.78	4.79E-06
rs2112945	5	91,322,882	*ARRDC3*	Intergenic	G	A	8.23	3.33	20.37	5.14E-06
rs9293595	5	91,308,742	*ARRDC3*	Intergenic	G	A	8.20	3.32	20.27	5.17E-06
rs10473955	5	91,308,785	*ARRDC3*	Intergenic	C	G	8.22	3.32	20.34	5.18E-06
rs35923949	5	91,305,127	*ARRDC3*	Intergenic	D	I	8.28	3.33	20.55	5.20E-06
rs10462501	5	91,325,596	*ARRDC3*	Intergenic	G	C	8.28	3.34	20.55	5.21E-06
rs9293594	5	91,304,870	*ARRDC3*	Intergenic	G	T	8.19	3.31	20.25	5.23E-06
rs34635591	5	91,327,949	*ARRDC3*	Intergenic	D	I	8.33	3.35	20.75	5.26E-06
rs7356540	5	91,303,719	*ARRDC3*	Intergenic	C	T	8.17	3.31	20.18	5.27E-06
rs1319728	5	91,337,995	*ARRDC3*	Intergenic	T	C	8.49	3.38	21.34	5.35E-06
rs10076564	5	91,304,952	*ARRDC3*	Intergenic	T	A	8.13	3.30	20.06	5.36E-06
rs7735328	5	91,306,578	*ARRDC3*	Intergenic	G	C	8.12	3.29	20.02	5.38E-06
rs1946485	5	91,321,142	*ARRDC3*	Intergenic	T	G	8.00	3.26	19.60	5.48E-06
rs9293596	5	91,324,764	*ARRDC3*	Intergenic	C	T	8.06	3.28	19.82	5.53E-06
rs12697821	5	91,341,328	*ARRDC3*	Intergenic	G	A	8.56	3.39	21.61	5.54E-06
rs12697822	5	91,341,783	*ARRDC3*	Intergenic	C	G	8.57	3.39	21.64	5.59E-06
rs1816089	5	91,313,509	*ARRDC3*	Intergenic	G	A	8.17	3.30	20.23	5.62E-06
rs1345786	5	91,314,004	*ARRDC3*	Intergenic	A	G	8.19	3.30	20.32	5.62E-06
rs10514351	5	91,296,544	*ARRDC3*	Intergenic	A	G	7.99	3.24	19.69	6.33E-06
rs28849408	5	91,352,859	*ARRDC3*	Intergenic	T	C	8.56	3.37	21.77	6.44E-06
rs193202648	3	153,825,916	*ARHGEF26-AS1*	Intronic	T	C	24.54	6.07	99.24	7.17E-06
rs10039163	5	91,375,152	*ARRDC3*	Intergenic	C	G	8.39	3.30	21.33	7.96E-06
rs16870354	5	91,317,587	*ARRDC3*	Intergenic	G	A	6.79	2.91	15.83	9.19E-06
rs7962430	12	31,047,274	*TSPAN11*	Intergenic	A	C	6.99	2.95	16.57	9.96E-06

rsID, variant identifier (dbSNP); chr, chromosome; OR, odds ratio; CI, confidence interval; P, uncorrected *p*-value

[1] Location on genome build GRCh37/hg19

[2] Odds ratio above 1 indicates an increased risk of eGFR reduction when carrying the tested allele, and below 1 a decreased risk

Table S5: 35 genetic variants suggestively associated ($p < 10^{-5}$) with lowest magnesium plasma level after platinum

rsID	Chr	Position [1]	Nearest gene	Consequence	Effect allele	Non-effect allele	Coef [2]	95 CI (lower)	95 CI (upper)	P
rs563097889	5	34,754,017	*RAI14*	Intronic	I	D	-0.06	-0.08	-0.04	5.16E-07
rs8081007	17	77,989,580	*TBC1D16*	Intronic	C	T	-0.06	-0.09	-0.04	9.06E-07
rs41301878	17	78,403,906	*ENDOV*	Stop gained	G	C	-0.09	-0.13	-0.05	3.74E-06
rs1659087	5	148,719,044	*AFAP1L1*	Intronic	G	T	-0.05	-0.07	-0.03	4.08E-06
rs41301876	17	78,403,801	*ENDOV*	Intronic	A	G	-0.09	-0.13	-0.05	4.17E-06
rs146564381	11	106,745,710	*GUCY1A2*	Intronic	D	I	0.08	0.05	0.11	4.18E-06
rs13250348	8	18,573,464	*PSD3*	Intronic	G	A	0.08	0.05	0.12	4.61E-06
rs4074023	17	77,976,135	*TBC1D16*	Intronic	A	G	0.04	0.03	0.06	5.04E-06
rs901729	8	18,565,805	*PSD3*	Intronic	A	G	0.09	0.05	0.13	5.33E-06
rs117467662	20	62,006,393	*CHRNA4*	Intronic	A	G	0.18	0.10	0.25	5.72E-06
rs150815093	20	62,011,140	*CHRNA4*	Intergenic	A	G	0.18	0.10	0.25	6.06E-06
rs71362773	18	75,544,345	*GALR1*	Intergenic	T	C	0.06	0.03	0.08	6.68E-06
rs6821388	4	146,186,595	*OTUD4*	Intergenic	T	C	0.10	0.06	0.14	6.76E-06
rs34289666	4	146,184,293	*OTUD4*	Intergenic	T	C	0.10	0.06	0.14	6.87E-06
rs4074022	17	77,976,421	*TBC1D16*	Intronic	G	C	0.05	0.03	0.07	6.88E-06
rs35764548	5	148,711,958	*AFAP1L1*	Intronic	I	D	-0.05	-0.07	-0.03	7.07E-06
rs13139038	4	146,183,902	*OTUD4*	Intergenic	C	T	0.10	0.06	0.14	7.18E-06
rs142367260	20	62,004,587	*CHRNA4*	Intronic	T	C	0.18	0.10	0.25	7.34E-06
rs2464645	10	56,698,996	*PCDH15*	Intronic	T	C	-0.06	-0.09	-0.03	7.50E-06
rs2444836	10	56,698,924	*PCDH15*	Intronic	C	G	-0.06	-0.09	-0.03	7.52E-06
rs1733757	10	56,699,960	*PCDH15*	Intronic	T	G	-0.06	-0.08	-0.03	7.68E-06
rs35295729	18	75,541,609	*MBP*	Intergenic	T	C	0.05	0.03	0.08	7.74E-06
rs35950653	14	30,553,458	*PRKD1*	Intergenic	A	G	0.15	0.09	0.21	8.12E-06
rs1304468	10	56,701,762	*PCDH15*	Intronic	C	T	-0.06	-0.08	-0.03	8.23E-06
rs813035	5	148,720,087	*AFAP1L1*	3' UTR variant	T	G	-0.05	-0.06	-0.03	8.45E-06
rs1659117	5	148,716,422	*AFAP1L1*	Intronic	G	A	-0.05	-0.06	-0.03	8.45E-06
rs352349	5	148,722,406	*AFAP1L1*	Intergenic	T	C	-0.05	-0.06	-0.03	8.55E-06
rs352350	5	148,721,551	*AFAP1L1*	Intergenic	C	G	-0.05	-0.06	-0.03	8.57E-06
rs3098389	5	148,713,163	*AFAP1L1*	Intronic	C	G	-0.05	-0.06	-0.03	8.61E-06
rs7240269	18	75,539,117	*GALR1*	Intergenic	C	G	0.05	0.03	0.08	8.61E-06
rs13102867	4	146,180,411	*OTUD4*	Intergenic	A	T	0.10	0.06	0.14	8.74E-06
rs117056236	1	170,141,999	*METTL11B*	Intergenic	C	T	0.11	0.06	0.15	9.02E-06
rs13133465	4	146,192,618	*OTUD4*	Intergenic	G	C	0.10	0.06	0.14	9.28E-06
rs8070465	17	77,978,990	*TBC1D16*	Intronic	G	T	0.05	0.03	0.07	9.35E-06
rs13146714	4	146,179,152	*OTUD4*	Intergenic	C	T	0.10	0.06	0.14	9.56E-06

rsID, variant identifier (dbSNP); chr, chromosome; coef, coefficient; CI, confidence interval; P, uncorrected *p*-value

[1] Location on genome build GRCh37/hg19

[2] Coefficient below zero represents an increased risk of hypomagnesemia when carrying the tested allele, and above zero a decreased risk

Table S6: 1 genetic variant suggestively associated ($p < 10^{-5}$) with hypomagnesemia (coded according to CTCAE-AKI v4.03 and need for magnesium suppletion) after platinum

**rsID**	**Chr**	**Position [1]**	**Nearest gene**	**Consequence**	**Effect allele**	**Non-effect allele**	**OR [2]**	**95 CI (lower)**	**95 CI (upper)**	**P**
rs6496125	15	96,135,977	*LINC00924*	Intergenic	C	T	13.32	4.29	41.37	7.54E-06

rsID, variant identifier (dbSNP); chr, chromosome; OR, odds ratio; CI, confidence interval; P, uncorrected *p*-value

[1] Location on genome build GRCh37/hg19

[2] Odds ratio above 1 indicates an increased risk of hypomagnesemia when carrying the tested allele, and below 1 a decreased risk

Table S7: Results of genetic association analyses of five candidate variants

Gene	Variant	Chr	BP	Effect allele	Non-effect allele	eGFR decline phenotype (linear regression)				CTCAE Acute Kidney Injury phenotype (logistic regression)			
						coef	95% CI		P	OR	95% CI		P
SLC22A2	rs316019	6	160,670,282	A	C	-0.005	-0.048	0.038	0.827	0.993	0.405	2.434	0.987
ERCC1	rs11615	19	45,923,653	G	A	0.027	-0.003	0.056	0.081	0.760	0.396	1.461	0.411
ERCC2	rs13181	19	45,854,919	G	T	-0.005	-0.035	0.024	0.720	0.590	0.306	1.138	0.116
ERCC1	rs3212986	19	45,912,736	A	C	0.027	-0.008	0.061	0.129	0.641	0.290	1.417	0.272
ERCC2	rs1799793	19	45,867,259	T	C	-0.004	-0.034	0.027	0.821	0.692	0.352	1.363	0.287

Chr, chromosome; BP, base pair position on genomic build GRCh37/hg19; MAF, minor allele frequency; coef, coefficient; 95% CI, 95% confidence interval; P, *p*-value; OR, odds ratio
